# Role of high resolution MR in assessment of cervical uterine carcinoma: staging, treatment planning and correlation with histopathology findings

**DOI:** 10.1186/1470-7330-15-S1-P43

**Published:** 2015-10-02

**Authors:** MF Grana, M Nazar, F Saguier, M Di Cecco, F Troncoso, E Eyheremendy, S De Luca, L Tolkachier, M Wirtz

**Affiliations:** 1 Hospital Aleman, Buenos Aires, Argentina

## Learning objectives

Review HRMRI techniques for pelvic evaluation of cervical uterine carcinoma using phased-array coil.

Discuss the accuracy of high resolution MR for diagnosis and staging of cervical uterine carcinoma

Discuss the correlation between MR findings, FIGO staging and treatment strategy

Discuss the correlation between MR features and histopathology findings in resected tumours

## Content organisation

• Uterine cervical carcinoma

• FIGO staging

• High resolution MRI

∘ Dedicated protocol

∘ Primary tumour detection

∘ Myometrial invasion

∘ Lymph node involvement

∘ Parametrial invasion

∘ Bladder and rectal invasion

∘ Vaginal involvement.

• Treatment strategies

• Histopathathology correlation

## Conclusion

Cervical cancer remains a major threat to women's health worldwide. MR is the imaging modality of choice to depict the primary tumour and assess local extent. Comparing the radiological findings with the postoperative histological reports, high resolution MR with a dedicated protocol demonstrated to be useful for primary tumour detection and for the assessment of myometrium invasion, lymph node commitment, parametrium invasion, bladder and rectal infiltration and vaginal involvement. This ability of MRI to demonstrate accurately the local extension of the tumour in patients with cervical cancer has become a useful tool to identify prognostic risk factors such as the depth of the infiltration, the tumour volume and the commitment of the adjacent structures. A correct evaluation of these factors is crucial for choosing and planning the most appropriate treatment.

